# Special Issue on Remote Sensing of Ocean Color: Theory and Applications

**DOI:** 10.3390/s20123445

**Published:** 2020-06-18

**Authors:** Trevor Platt, Shubha Sathyendranath, Heather Bouman, Carsten Brockmann, David McKee

**Affiliations:** 1 Plymouth Marine Laboratory, Plymouth PL1 3DH, UK; ssat@pml.ac.uk; 2 Department of Earth Sciences, University of Oxford, Oxford OX1 3AN, UK; heather.bouman@earth.ox.ac.uk; 3 Brockmann Consult GmbH, 21029 Hamburg, Germany; carsten.brockmann@brockmann-consult.de; 4 Department of Physics, University of Strathclyde, Glasgow G4 0NG, UK

## Abstract

The editorial team are delighted to present this Special Issue of *Sensors* focused on Remote Sensing of Ocean Color: Theory and Applications. We believe that this is a timely opportunity to showcase current developments across a broad range of topics in ocean color remote sensing (OCRS). Although the field is well-established, in this Special Issue we are able to highlight advances in the applications of the technology, our understanding of the underpinning science, and its relevance in the context of monitoring climate change and engaging public participation.

## 1. Introduction

Although the title refers to “ocean color”, it is important to acknowledge that the field implicitly includes freshwater systems which share many of the optical challenges of coastal and shelf seas, often in an amplified manner. This is reflected in [1,2], which report on current efforts to resolve water quality parameters in optically complex freshwater and marine systems. It is also important to emphasize that a key attribute of ocean color remote sensing (OCRS) is the unique ability to provide vital insights into biological processes in surface waters, with [3,4] both highlighting links between the physical and biological processes impacting on optical signals. The link between inherent optical properties and OCRS signals is further explored in [5], illustrating that there is still room for further progress in fully appreciating the radiative transfer processes that form remote sensing signals in the aquatic environment. Our understanding of the relationships between optical and biogeochemical parameters is inevitably influenced by the choice of statistical methods, and [6] reveals the impact of choosing either type I or II regressions on derived bio-optical relationships.

As the technology available to the community continues to progress, new opportunities emerge. The development of geostationary OCRS has the potential to revolutionize the monitoring of tidal, diurnal and other short-term episodic events. Data quality is, however, absolutely vital, and in [7] we are presented with the challenges and latest developments in the geometric correction of GOCI images.

The context for all of our work observing aquatic systems from space is the impact of anthropogenic pollution and climate change. The topic of monitoring direct pollution events is addressed in [8], which presents new optical modeling that suggests a route to use OCRS to monitor oil pollution at the sea surface. The global climate emergency presents a longer-term challenge. OCRS has been identified as an Essential Climate Variable by the World Meteorological Organisation. The provision of continuous global coverage over multiple decades that is consistent between generations of satellite sensors is a major challenge that is addressed in [9]. The perspective of long timescales over which the effects of climate change will be observed has also brought renewed focus on historical data sets. The earliest ocean color observations were made in the nineteenth century using simple Secchi disks and chemical color standards (the Forel Ule scale), and the resulting historic data sets provide a vital gateway to establish long-term changes in ocean color. In an age of ever-increasing technological complexity, there is a wonderful charm to presenting citizen scientists with an affordable means of making a meaningful contribution to the monitoring of our natural water systems [10]. As climate change will undoubtedly affect every human being that shares the planet, it is ever more essential that opportunities are available for citizens to contribute to all aspects of the challenge.

This special issue was the brainchild of Trevor Platt. He was the driving force in assembling the editorial team, promoting the issue within the community and providing editorial oversight to all of the contributing papers. As was the case throughout his long and distinguished career, Trevor carried out this role with great technical rigor, meticulous attention to detail and with the ambition to make a significant contribution to the field. He gave his time generously and was unfailing in his dedication to supporting friends and colleagues across the community, especially early-career researchers. Trevor has left an indelible footprint on our field not only through his outstanding achievements in research but also in his service to the scientific community through his steadfast commitment to international teaching, capacity building and science coordination. Very sadly this was to be one of Trevor’s final contributions to his beloved field of research, as he passed away on 6 April 2020 as this Special Issue neared completion. His fellow editors would like to dedicate this Special Issue to his memory as a founding pioneer of the field, a supportive mentor and, most importantly, as a cherished friend. He will be sadly missed.
